# Outcomes of Gestational Trophoblastic Disease Management: A Single Centre Review

**DOI:** 10.3390/medicina59091632

**Published:** 2023-09-08

**Authors:** Eva Pavla Malovrh, Nuša Lukinovič, Monika Sobočan, Jure Knez

**Affiliations:** 1 Faculty of Medicine, University of Maribor, 2000 Maribor, Slovenia; evapavla.malovrh@student.um.si (E.P.M.); nusa.lukinovic@student.um.si (N.L.); jure.knez@ukc-mb.si (J.K.); 2 Division of Gynaecology and Perinatology, University Medical Centre Maribor, 2000 Maribor, Slovenia

**Keywords:** gestational trophoblastic disease, molar pregnancy, hCG, treatment outcomes

## Abstract

*Background and Objectives*: Gestational trophoblastic disease (GTD) is a group of pregnancy-related malignant and premalignant diseases. The aim of this study was to assess the prognostic value of clinical characteristics to predict treatment outcomes in women with GTD. *Materials and Methods*: In this retrospective study, 34 patients treated for GTD at the Division of Gynaecology and Perinatology, University Medical Centre Maribor, between 2008 and 2022 were identified. Clinical and pathological characteristics were obtained by analysing patient data records. *Results*: Within the cohort of 34 patients with GTD, 29 patients (85.3%) had a partial hydatidiform mole (HM) and five patients545 (14.7%) had a complete HM. Two patients with a complete HM developed a postmolar gestational trophoblastic neoplasia (GTN), which represents 5.8% of all cases. *Conclusions*: GTD is a rare disease that is frequently asymptomatic. The subsequent consequences of GTD, which can lead to malignant transformation, as well life-threatening disease complications, warrant training for early recognition of HMs and timely treatment and surveillance.

## 1. Introduction

Gestational trophoblastic disease (GTD) is a heterogeneous group of rare disorders that are characterised by abnormal proliferation of trophoblastic tissue. It encompasses premalignant partial (PHM) and complete hydatidiform moles (CHMs), as well a malignant diseases such as malignant invasive moles, choriocarcinomas, placental-site trophoblastic tumours (PSTTs) and epithelioid trophoblastic tumours (ETTs) [1]. The malignant trophoblastic disorders are collectively known as gestational trophoblastic neoplasia (GTN) [2]. These entities may arise after a normal pregnancy, miscarriage, or ectopic pregnancy. Most GTNs arise after complete molar pregnancies [3].

The hydatidiform mole (HM) is the most common type of GTD. Approximately 80% of GTDs are HMs [1,4]. Epidemiological studies show a wide variation in the incidence of HMs. In most parts of the world, the incidence of HMs is 1 per 1000 pregnancies. In high-income countries, the incidence of CHMs is approximately 1–3 per 1000 pregnancies, and the incidence of PHMs is about 3 per 1000 pregnancies [2]. Higher frequencies of HM have been reported in patients of Asian origin with rates ranging from 1 to 10 per 1000 pregnancies [1]. Differences in incidence can partially be attributed to varying diagnostic criteria, reporting practices, quality of epidemiologic data, and diet [4].

HM appears more frequent at the extremes of reproductive age (<15 and >45 years). Young women have a two-fold risk of having a molar pregnancy. The risk increases again after age 35, and there is a 5–10 times increased risk after 45 years [2]. There is an increasing risk for CHM with advancing maternal age. This is thought to be associated with abnormal gametogenesis and fertilisation of the ovum produced at extremes of reproductive age [2,5]. History of a previous molar pregnancy increases the risk to 10–20 times in comparison with the general population [4].

The classic clinical signs of GTD are vaginal bleeding, uterine enlargement greater than expected for gestational age, absent foetal heart tones, higher-than-expected level of human chorionic gonadotropin (hCG), theca-lutein cysts, hyperemesis, preeclampsia, and hyperthyroidism [6]. However, many patients are asymptomatic at diagnosis due to wide use of ultrasound in early pregnancy [1,2,7]. Diagnosis is usually made in the first trimester with clinical signs, ultrasound imaging, and hCG levels testing, and it is confirmed with histological examination of evacuated tissue [2,8,9]. Additional imaging, such as chest X-ray, CT, or MRI, can be used for staging of GTD [1,9].

Standard treatment of HMs is surgical, usually with suction dilation and curettage, preferably performed under ultrasound guidance. For women who are older or do not wish to preserve fertility, hysterectomy is considered as an alternative [10]. After surgical management and confirmation of the diagnosis, patients are monitored with quantitative serum hCG values until they are in the normal range [6]. An increase or plateau of hCG levels indicates a post-molar GTN [1]. In the case of a suspected GTN, the diagnosis is confirmed according to the FIGO criteria [2] for diagnosis of postmolar gestational neoplasia. The primary treatment for most forms of GTN is chemotherapy, although surgical intervention may be required for some cases and for management of complications [2,9]. Low-risk GTN (FIGO/WHO score 0–6) is treated with single-agent chemotherapy, and high-risk GTN (FIGO/WHO score >6) is treated with multi-agent chemotherapy [1].

The aim of this study was to evaluate the impact of clinical presentation, patient characteristics, and treatment on outcomes in patients with GTD.

## 2. Materials and Methods

In this single-centre retrospective study, the patients were analysed through a database analysis of all pregnant women who were referred to the University Medical Centre Maribor (UMC Maribor) between 2008 and 2022 and diagnosed with GTD upon histopathological tissue examination. Prior to their treatment, all patients signed a written informed consent form to allow the use of their medical records retrospectively for research purposes. This study was approved by the National Ethics Committee, reg. no. 0120-394/2021/6.

### 2.1. Patient Selection

**Figure 1:**
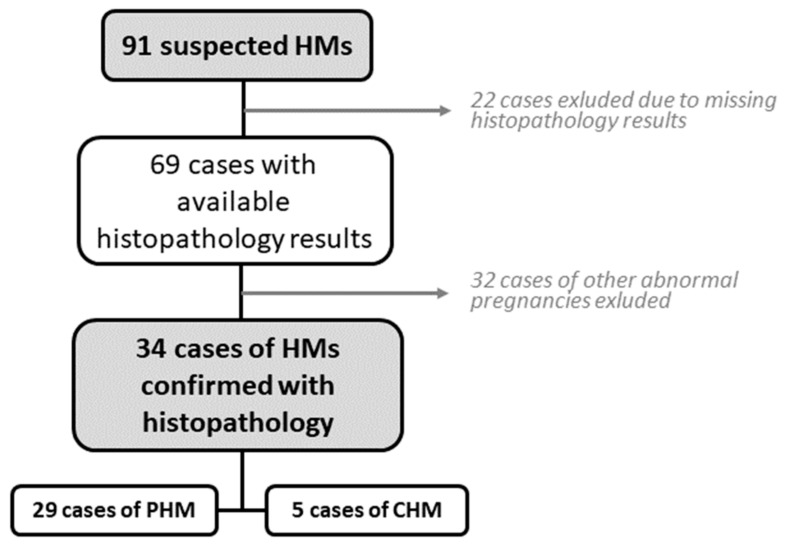
Patient selection process.

After initial screening, we retrospectively analysed the data of 91 patients admitted to UMC Maribor as suspected molar pregnancies. A total of 22 cases were initially excluded due to essential clinical patient data missing from the electronic records. One patient with GTN was also excluded, due to data about a preceding molar pregnancy treatment performed at the regional hospital, with essential data unavailable. Of the remaining 69 patients, 32 were excluded as histopathology confirmed benign pathology such as failed pregnancies (missed abortions) without GTD. Ultimately, 34 cases of confirmed molar pregnancies by histopathology were found and included in statistical analysis (Figure 1).

There were 3 cases of gestational trophoblastic neoplasia in which antecedent pregnancy was not molar (spontaneous abortion, ectopic pregnancy, and normal term pregnancy, respectively). Those 3 cases were also excluded from statistical analysis; however, they are studied separately in this manuscript.

### 2.2. Analysed Clinical Features

The analysed clinical features were patient age at time of first GTD diagnosis, symptoms, gestational age, gynaecological examination, hCG levels at time of first diagnosis, date of treatment initiation, treatment method, postoperative histopathological analysis, reproductive history (antecedent pregnancies, preceding labours, previous spontaneous abortions), the type of conception (spontaneous or assisted reproductive techniques), ultrasound characteristics at time of presentation, data on management approach, and hCG assessments.

### 2.3. Statistical Analysis

We analysed demographic data through descriptive statistics. Qualitative variables were described with absolute and relative (percentage) frequencies. Quantitative variables were summarized with the means (standard deviations, SD) and medians (minimum, maximum) in case of normal and non-normal distribution, respectively. CHMs and PHMs were compared with chi-squared or Fisher exact test for the qualitative variables and Student’s *t*-test or Mann–Whitney test for normal and non-normal quantitative variables, respectively. A *p*-value <0.05 was considered as significantly different. All statistical analyses were performed using SPSS Version 26.0 (IBM Corp., Armonk, NY, USA).

## 3. Results

A total of 34 patients, aged from 18 to 53 years old, were included in our study. The median age of patients at the time of diagnosis was 31 years (range 18–53). Of 34 patients with molar pregnancy, 29 patients (85%) had PHM, and five patients (15%) had CHM (CI: 1.73–1.98) (Table 1). In patients with PHM, the median uterine size was 103 mm (min. 71 mm, max. 130 mm) (n = 14), and in patients with a complete HM, median uterine size was 93 mm (min. 89 mm, 175 mm) (n = 4). The median gestational age at time of diagnosis was 10 weeks (min. 6, max. 13). The median gestational age in PHM was 10 weeks (min. 6 weeks, max. 13 weeks) (n = 29) at time of diagnosis and 9 weeks in CHM (min. 6 weeks, max. 11 weeks) (*p* = 0.743). Of 28 patients with PHM, 10 patients (34%) had a reproductive history of spontaneous miscarriage (CI: 0.22–0.57). Among those with CHM, the history of spontaneous miscarriage was positive in three patients (60%) and negative in two (40%). In patients with PHM, 34% (n = 10) were nulliparous, 45% had given birth once, and 17% (n = 5) had given birth two or more times (CI: 0.52–1.01). In patients with CHM, 60% (n = 3) had given birth once, and two patients were nulliparous (CI: 0.52–1.01). Of all cases of HM, 94% (n = 32) were conceived naturally and 6% (n = 2) were conceived using assisted reproduction techniques (CI: 0.98–1.14).

**Figure 2:**
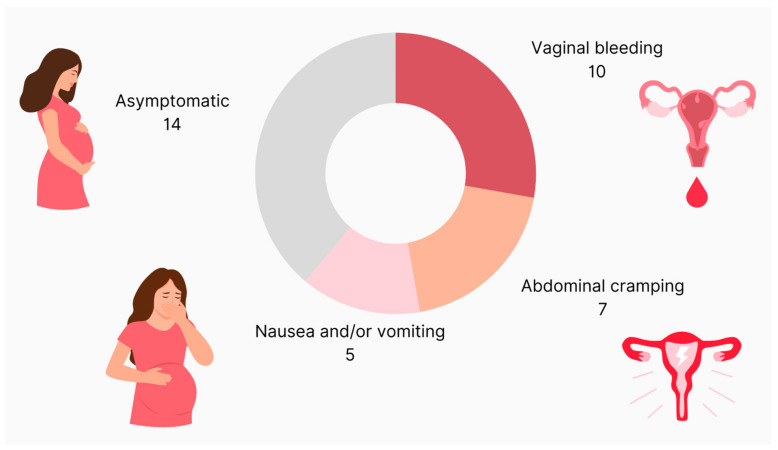
Clinical presentation of patients with molar pregnancy.

At the time of diagnosis, 41% of patients (n = 14) had no symptoms, 6% (n = 2) had cramping only, 15% (n = 5) complained of nausea and/or vomiting, another 15% (n = 5) had only vaginal bleeding and another 15% (n = 5) had cramping as well as vaginal bleeding (CI: 0.93–2.10) (Figure 2).

In three patients, the report on symptoms was missing.

The value of hCG at the time of diagnosis was observed. The median hCG was 44.559 (min. 40 mIU/L, max. 909.000 mIU/L).

**Figure 3:**
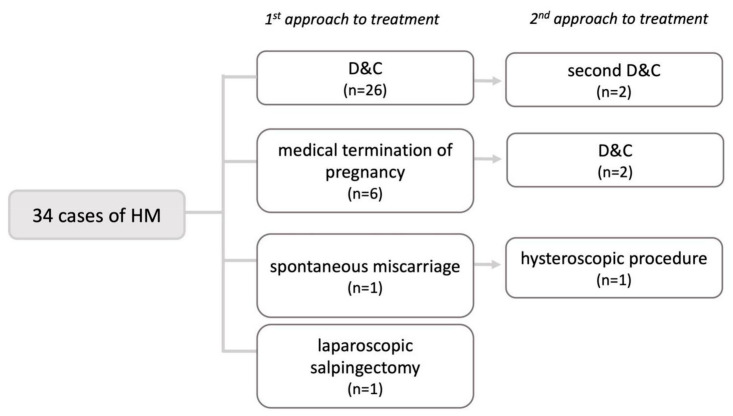
Means of pregnancy termination in patients with molar pregnancy. HM—hydatidiform mole, D&C—dilation and curettage.

Twenty-six patients (77%) underwent a dilation and curettage (D&C); in six patients (18%), pregnancy was terminated medically (two of them because they did not consent to D&C, and the other four because molar pregnancy was not suspected and they were treated as a missed abortion), one patient (3%) had a spontaneous miscarriage, and one patient (3%) underwent a laparoscopic salpingectomy due to a tubal ectopic pregnancy (CI: 1.08–1.56) (Figure 3).

After initial treatment, 26 patients (77%) had no complications, three patients (9%) had vaginal bleeding, and five patients (15%) had retained products (CI: 0.17–0.89). A total of 24 patients (71%) did not need additional treatment, while 10 patients (29%) did (CI: 0.13–0.46).

Five patients needed additional surgical treatment. Two patients (6%) underwent a second D&C. Another two patients (6%) had their first D&C as a second form of treatment after an initial medical termination of pregnancy. One patient (3%) underwent hysteroscopy after a spontaneous miscarriage (CI: 0.17–0.89).

The first follow-up after treatment was 17.3 ± 13.61 days. The median value of the follow-up hCG measurement was 592.00 (min. 0, max. 126,700 IU/mL). The median number of days to hCG negativization was 69 days (CI: 54–79). Two patients developed post-molar GTN (an invasive mole each), which represents 6% of all cases (CI: 0.02–0.14). Both of these cases occurred after diagnosis of CHM.

### Cases of GTN

During the patient selection process, four other GTNs were found. Because they did not originate from molar pregnancy, they were not included in the statistical analysis. However, there were three cases of post-molar GTN originating from a complete mole. In one case, GTN was diagnosed 93 days (13 weeks and 2 days) after evacuation of molar tissue. In the other two cases, GTN was diagnosed in less than 4 weeks after D&C (18 days in patient no. 3 and 23 days in patient no. 4) (Table 2). Overall, there were six cases of GTN. Clinical characteristics of patients with GTN are presented in Table 2. In addition to four invasive moles, there was one case of PSTT, which developed after normal pregnancy. Additionally, we identified one case of choriocarcinoma originating from an ovarian ectopic pregnancy, which was previously published [11].

## 4. Discussion

In our study, we have evaluated the clinical characteristics of women with gestational trophoblastic disease. We have demonstrated that PHM is significantly more common compared to CHM; however, the risk of developing GTN is higher in women with CHM. We did not identify any clinical characteristics that would allow for more accurate prediction of developing GTN. However, since GTD is a rare disease, our analysis is limited by the small sample size.

Within this study, PHMs were more frequent (85.3%) than CHMs (14.7%) (CI: 1.73–1.98). This correlates with results from a similar, previously published study by Lelic et al., which found 93.5% of cases were PHM and 6% of cases were CHM, with one case (0.5%) undefined [12]. Other similar studies described a more equal distribution between the two subtypes of HMs [3,13,14].

The diagnosis of HM is based on clinical presentation, transvaginal ultrasound imaging, and pathohistological examination of evacuated tissue. Nowadays, many patients are asymptomatic at the time of diagnosis due to the wide use of ultrasound scans in early pregnancy, and thus receive early diagnosis [15]. Joneborg et al. reported that vaginal bleeding was statistically more common in a group of patients from 1988 to 1993 than one from 1991 to 2010. They concluded that vaginal bleeding is still the most commonly presenting symptom. They also observed that, nowadays, patients are more often asymptomatic due to earlier diagnosis [14]. In line with their observations, a great proportion of our patients (41%) were also asymptomatic and, among those presenting with at least one symptom, the most common was vaginal bleeding.

Ultrasound is the initial imaging technique employed in the investigation of cases of molar pregnancy [16]. The advances of ultrasound imaging in recent decades have shifted the diagnosis of HMs from the second to the first trimester of pregnancy. Gestational age highly correlates with the accuracy of the ultrasound diagnosis of HM. The current literature suggests that the mean gestational age at diagnosis is at present around 10–12 weeks [6], which correlates with our findings. The median gestational age at the time of diagnosis in our study was 10 weeks. Diagnosing PHM using TVUS is more challenging compared to CHM, due to sometimes less intensive ultrasound characteristics misleading (especially inexperienced) sonographers in their initial assessment [15]. Ultrasound appearance suggestive of a CHM is an enlarged uterus with a heterogeneous, primarily echogenic mass that has multiple hypoechoic foci (appearing as a snowstorm), as well as numerous small, anechoic cystic spaces that range in size from 1 to 30 mm (described as a cluster of grapes). There may be multiple large, bilateral, functional ovarian cysts that are theca lutein cysts. No foetal parts are present, except in the extremely rare case of a CHM with a coexisting diploid twin. Subtler US changes are characteristic of a PHM. Before 10 weeks of gestation, hydropic changes are frequently not apparent. It is common to see a placenta that is larger than the uterine cavity and has internal cystic changes that create a “Swiss cheese pattern.” Theca lutein cysts are uncommon. Foetal parts are present as amorphous echoes. If a foetus is formed, it typically has a range of severe abnormalities [17]. In a cohort study of 295 women, US imaging diagnosed a significantly (*p* < 0.001) higher number of CHMs (74.2%) than PHMs (40.7%) [16]. This is largely due to subtler US changes in PHMs compared to CHMs. Another reason is that PHMs tend to grow more slowly, and some of their characteristics may not be visible at the earlier stage [6]. Overall, the specificity of ultrasound for accurately predicting hydatidiform mole is less than 50% [15,18,19]. However, detection rates are higher for CHMs than PHMs, and increase after 14 weeks of gestation.

Despite the pathologic and clinical differences, the management of patients with CHMs and PHMs is similar. Suction D&C is the preferred method of evacuation for most patients who wish to preserve fertility. We found that 77% of patients in our study had a D&C. However, in 18% of cases, pregnancy was terminated medically. We hypothesize that this is true in cases where HM was not initially suspected. This finding accentuates the importance of correct ultrasound diagnoses. With medical methods, there is a higher rate of incomplete molar tissue removal and a higher chance that an HM would stay unrecognised [18,19]. Women who have an unrecognised molar pregnancy and undergo medical or surgical abortion are at increased risk of life-threatening complications of GTN, and require more surgical intervention and chemotherapy [18,19]. The histological assessment of material obtained from the medical or surgical management of all miscarriages is recommended to exclude GTN if no foetal parts are identified at any stage of the pregnancy [20,21,22].

After the evacuation of molar tissue, follow-up hCG monitoring is essential for early detection of GTN. Currently, FIGO recommends hCG monitoring every 1–2 weeks until there is a normal level, followed by a single confirmatory normal hCG level in a month for PHMs and monthly normal hCG levels for 6 months for CHMs [2,6]. After normalisation of hCG has been confirmed with a second hCG value, the risk of developing postmolar GTN is extremely small [6]. However, there is a rare group of patients in whom HM appears to undergo spontaneous resolution, with normalisation of serum hCG, but subsequently relapse and develop GTNs [23]. In our study, the median number of days to beta HCG negativisation was 68.5 days (95% CI [54–79]), which represents approximately 10 weeks. According to literature, the mean time to hCG normalisation in case of PHMs is 6 weeks and, in case of CHMs, 7 weeks [24].

In most patients, HMs regress spontaneously after evacuation of molar pregnancy, but in approximately 15–20% of CHMs and 0.5–1% PHMs, trophoblastic tissue remains active, leading to a sustained rise or plateau in hCG levels. This indicates the need for evaluation and treatment for GTNs [25]. Definition of a post-molar GTN is based on hCG level changes as defined by the FIGO criteria [2]. For monitoring of GTNs, an hCG assay that can detect all forms of hCG should be used when possible because trophoblastic tumours can secrete different hCG subtypes [26]. Although rare instances of long latent periods preceding postmolar GTNs have been reported, almost all episodes of malignant sequelae occur within 6–12 months of evacuation [6]. This correlates with our results with all three cases of post-molar GTNs occurring in less than 6 months after D&C.

Overall, there were six cases of GTNs. We recorded four cases of an invasive mole, one case of a PSTT, and one choriocarcinoma. Only three cases originated from molar pregnancy. One invasive mole occurred after spontaneous miscarriage, while the PSTT occurred after normal-term pregnancy and the choriocarcinoma followed an ectopic pregnancy treated with methotrexate. In three cases, hCG at the time of diagnosis was more than 225.000 IU/L, which is above measurable threshold.

Hysterectomy was required in three patients, two of which were still of reproductive age. The patient with choriocarcinoma underwent right-sided laparoscopic adnexectomy due to initially suspected tubal ectopic pregnancy. Two patients with invasive mole were treated with methotrexate only, without any surgical intervention. As for treatment outcomes, four patients went into complete remission, another two cases were resistant to primary chemotherapy, and then, after additional chemotherapy cycles, went into complete remission.

According to the FIGO 2021 recommendations [2], any GTN should be staged and scored according to the current FIGO staging and prognostic scoring system. While going through our cases patients’ records, we observed that information on the FIGO stage and WHO prognostic score was missing in some of them. In those cases, we reassessed patients’ clinical data and calculated their scores according to FIGO/WHO scoring system. In the 2000 FIGO staging and classification [2], a risk score of 6 and below is classified as low-risk, and above 6 is considered high-risk. Two patients classified as high-risk, with scores of 7 and 16, respectively. Four of our cases classified as low-risk, with scores of 1, 5, 6, and 6. Two low-risk cases were initially resistant to primary chemotherapy. These results build on continuing debate on whether low-risk patients scoring 5–6 should still be considered low risk and treated with single-agent chemotherapy. As most of the patients with prognostic scores 5–6 develop resistance to single-agent chemotherapy, there is a need to better identify those patients who can instead be treated with more intensive therapy from the outset [27]. While this challenge is yet to be resolved, clinicians should ensure they use the FIGO/WHO scoring system to facilitate the comparison of data.

The main limitations of our study were its retrospective nature, the retrieval of patient records over 15 years, and the low number of cases. Due to the small sample size, especially in the group of CHMs, interpretation of the results was limited, preventing us from drawing any statistically significant conclusions. We also could not access data from other regional hospitals that referred patients with post-molar GTNs to our institution.

## 5. Conclusions

Our study shows that molar pregnancy is a rare disease that is frequently asymptomatic. In rare cases, it can lead to subsequent development of a GTN with unfavourable complications, which underpins the importance of early accurate diagnosis. In our series, all GTN cases occurred after CHM, which has a much more characteristic ultrasound appearance compared to PHM. Hence, especially in these cases, correct diagnosis is likely to be of crucial importance for correct management and follow-up of patients.

## Figures and Tables

**Table 1:** Characteristics of patients with PHM vs. CHM.

Characteristic	Category	PHM	CHM	Significance
Number of patients	-	29 (85%)	5 (15%)	-
Age at time of diagnosis (median)	-	30	33	*p* > 0.205
History of prior miscarriage	Yes	10 (34%)	3 (60%)	*p* > 0.306
	No	18 (62%)	2 (40%)	
Parity prior to GTD diagnosis	0	10 (35%)	2 (40%)	*p* > 0.584
	1	13 (45%)	3 (60%)	
	2 or more	5 (17%)	0 (0%)	
Uterine size, mean ± SD	-	101.07 ± 16.39	112.50 ± 41.71	*p* > 0.266

**Table 2:** Characteristics of patients with GTN.

Categories	Patient No. 1	Patient No. 2	Patient No. 3	Patient No. 4	Patient No. 5	Patient No. 6
Age at diagnosis (years)	29	32	53	34	30	44
Antecedent gestational event	CHM	spontaneous abortion	CHM	CHM	normal pregnancy	ectopic pregnancy
Histological diagnosis of GTN	invasive mole	invasive mole	invasive mole	invasive mole	PSTT	choriocarcinoma
hCG at diagnosis (IU/L)	128.000	>225.000	>225.000	133.773	102	>225.000
Days until hCG negativisation	152	167	unknown	unknown	37	unknown
FIGO stage (I, II, III, IV, unknown)	I	I	I	I	I	IV
WHO prognostic scoring system, adapted by FIGO	6	6	7	5	1	16
Surgical treatment	hysteroscopic resection of polypoid mass	hysterectomy	hysterectomy	no surgical treatment	hysterectomy	right-sided laparoscopic adnexectomy
Chemotherapy	methotrexate	methotrexate	unknown	methotrexate	none received	cisplatin/etoposide
Number of chemotherapy cycles	7	7	unknown	unknown	not applicable	unknown
Outcome	Complete remission	Complete remission	Complete remission	Complete remission	Complete remission	Complete remission

## Data Availability

The data presented in this study are available on request from the corresponding author.
